# Supplementary material for: Phosphodiesterases 4B and 4D Differentially Regulate cAMP Signaling in Calcium Handling Microdomains of Mouse Hearts
Source: Cells. 2024 Mar 8;13(6):476. doi: 10.3390/cells13060476 (PMC10969065; doi:10.3390/cells13060476)
Supplement: Supplementary file 1 [file cells-13-00476-s001.zip › cells-2762295-supplementary.pdf]

SUPPLEMENTARY MATERIAL TO:

Phosphodiesterases 4B and 4D differentially regulate cAMP signaling in calcium handling microdomains of mouse hearts

Axel E. Kraft [1,2], Nadja I. Bork [1,2], Hariharan Subramanian [1,2], Nikoleta Pavlaki [1,2], Antonio V. Failla [3], Bernd Zobiak [3], Marco Conti [4,†] and Viacheslav O. Nikolaev [1,2,*,†]

1 Institute of Experimental Cardiovascular Research, University Medical Center Hamburg-Eppendorf, 20246 Hamburg, Germany
2 German Center for Cardiovascular Research (DZHK), Partner Site Hamburg/Kiel/Lübeck, 20246 Hamburg, Germany
3 UKE Microscopy Imaging Facility (UMIF), University Medical Center Hamburg-Eppendorf, 20246 Hamburg, Germany
4 Eli and Edythe Broad Center of Regeneration Medicine and Stem Cell Research, Department of Obstet-rics, Gynecology, and Reproductive Sciences, Center for Reproductive Sciences, University of California, San Francisco, CA 94143, USA; marco.conti@ucsf.edu
* Correspondence: v.nikolaev@uke.de; Tel.: +49-(0)-40-7410-51391
† These authors contributed equally to this work.

Detailed Methods

Cardiomyocyte Isolation

Mice at the age of 8-20 weeks were euthanized by cervical dislocation, the heart was rapidly explanted and washed in a petri dish with ice-cold PBS. It was mounted via the aorta onto a blunted 20 G cannula, which allows a retrograde perfusion at 3 mL/min with Ca^{2+}-free perfusion buffer (NaCl, 1.13 mmol/L; KCl, 47 mmol/L; $KH_2PO_4x2H_2O$, 6 mmol/L; $MgSO_4x7H_2O$, 12 mmol/L; $NaHCO_3$, 120 mmol/L; $KHCO_3$, 100 mmol/L; HEPES, 100 mmol/L; taurine, 300 mmol/L; glucose, 5.55 mmol/L; 2,3-butanedione monoxime (BDM), 9.89 mmol/L, pH 7.4) for 3 min. This was followed by 9 min of enzymatic digestion with perfusion buffer containing 0.04 mg/mL liberase DH (Sigma Aldrich), 0.0025% trypsin and 12.5 µmol/L $CaCl_2$. The atria were carefully excised and discarded, whereas the digested ventricles were dissected for 30 s in 2.5 mL digestion buffer. Enzymatic digestion was stopped, by adding 2.5 mL stop buffer 1 (perfusion buffer containing 1% FCS and 50 µM $CaCl_2$) to the cell suspension and was carefully homogenized for 3 min with a 1 mL syringe. After separating ventricular cardiomyocytes (CMs) from other cell types by gravitation, the supernatant was discarded and the cell pellet was resuspended in stop buffer 2 (perfusion buffer containing 0.5% FCS and 37.5 µmol/L $CaCl_2$). Cell suspension was recalcified in five steps, resulting in a final Ca^{2+} concentration of 1 mmol/L. CMs were plated onto laminin coated glass coverslides. Cells were incubated at 37°C and 5% CO_2 until needed for measurements.

FRET measurements

Glass coverslides with adherent CMs were mounted in a measuring chamber and washed once with FRET buffer (NaCl, 144 mmol/L; KCl, 5.4 mmol/L; $MgCl_2x7H_2O$, 1 mmol/L; $CaCl_2$, 1 mmol/L; HEPES, 10 mmol/L; pH 7.4). Fresh FRET buffer was then added to the chamber and imaging was performed using an inverted fluorescent imaging system build around Leica DMI 3000B microscope equipped with 63x oil immersion objective. The donor fluorophore was excited with a single wavelength LED of 440 nm using CoolLED device. To reduce photo bleaching to a minimum level, LED intensity and exposure time were selected to get a good signal-to-noise ratio. Fluorescent emission was split into CFP and YFP channels using DV2 DualView and images were captured using OptimMOS camera (both from Photometrics, Tucson, AZ, USA). Cells were treated with several pharmacological compounds isoproterenol (ISO, propranolol, 8-methoxymethyl-3-isobutyl-1-methylxanthine (IBMX) and forskolin. Donor (CFP) fluorescence was detected at 480±15 nm, acceptor (YFP) fluorescence at 535±20 nm. The spectral bleedthrough factor (detected donor fluorescence in the acceptor channel) was determined and the calculated acceptor/donor ratio was corrected.

PDE activity assay

PDE4B and PDE4D were immunoprecipitated from WT, PDE4B-KO and PDE4D-KO heart lysates, and PDE activity was measured as previously described, see Ref. 19.

Supplementary Figures

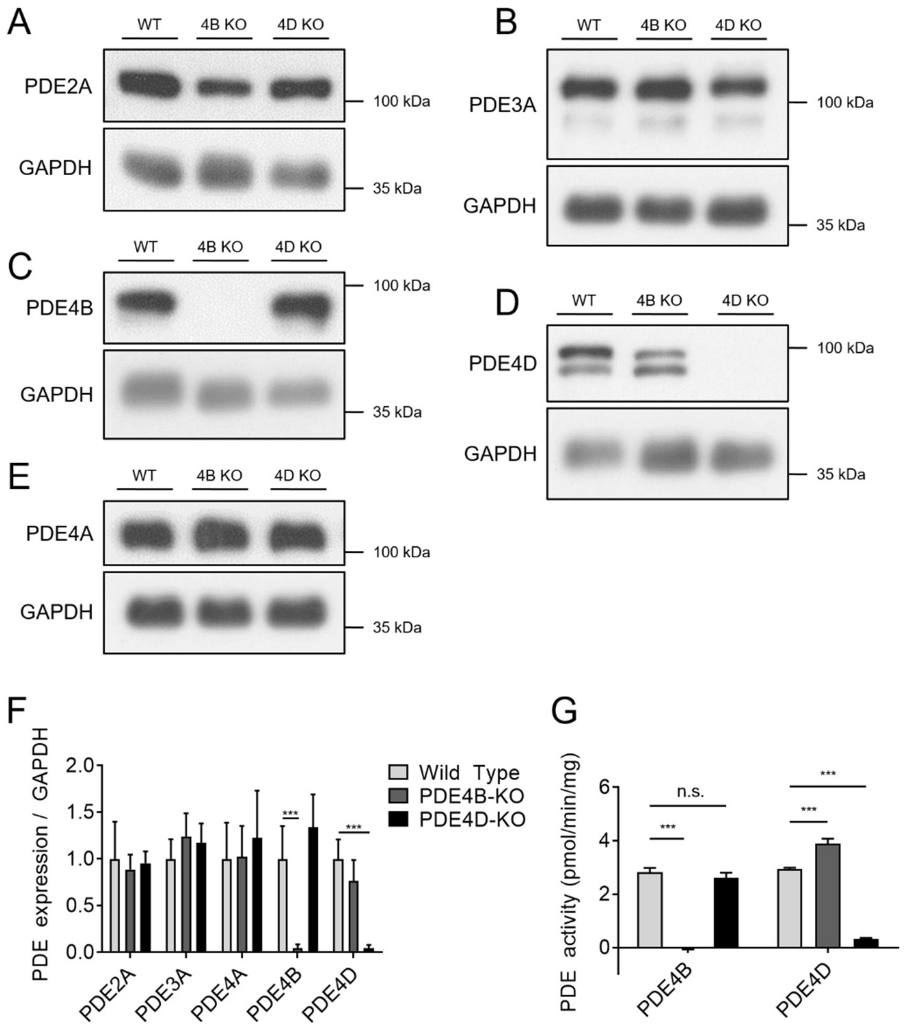

Supplementary Figure S1. Conserved expression of PDE2A, PDE3A, PDE4A and PDE4B/D in PDE4B and PDE4D deficient mice. (A-E) Representative western blots performed with 20 µg heart lysates each for **(A)** PDE2A, **(B)** PDE3A, **(C)** PDE4B, **(D)** PDE4D and **(E)** PDE4A in wildtype or PDE4B and PDE4D deficient mice. **(F)** Quantification of PDE expression. Data of 5 individual hearts are presented as means ± SEM. *** - significant differences at $p<0.001$ by one-way ANOVA followed by Sidak's multiple comparison test. **(F)** Catalytic activity of PDE4 measured in PDE4B and PDE4D immunoprecipitates obtained from WT, PDE4B-KO and PDE4D-KO hearts. Data are means ± SEM, n=3 each. n.s. – not significant, *** - significant differences at $p<0.001$, by one-way ANOVA followed by Sidak's multiple comparison test.

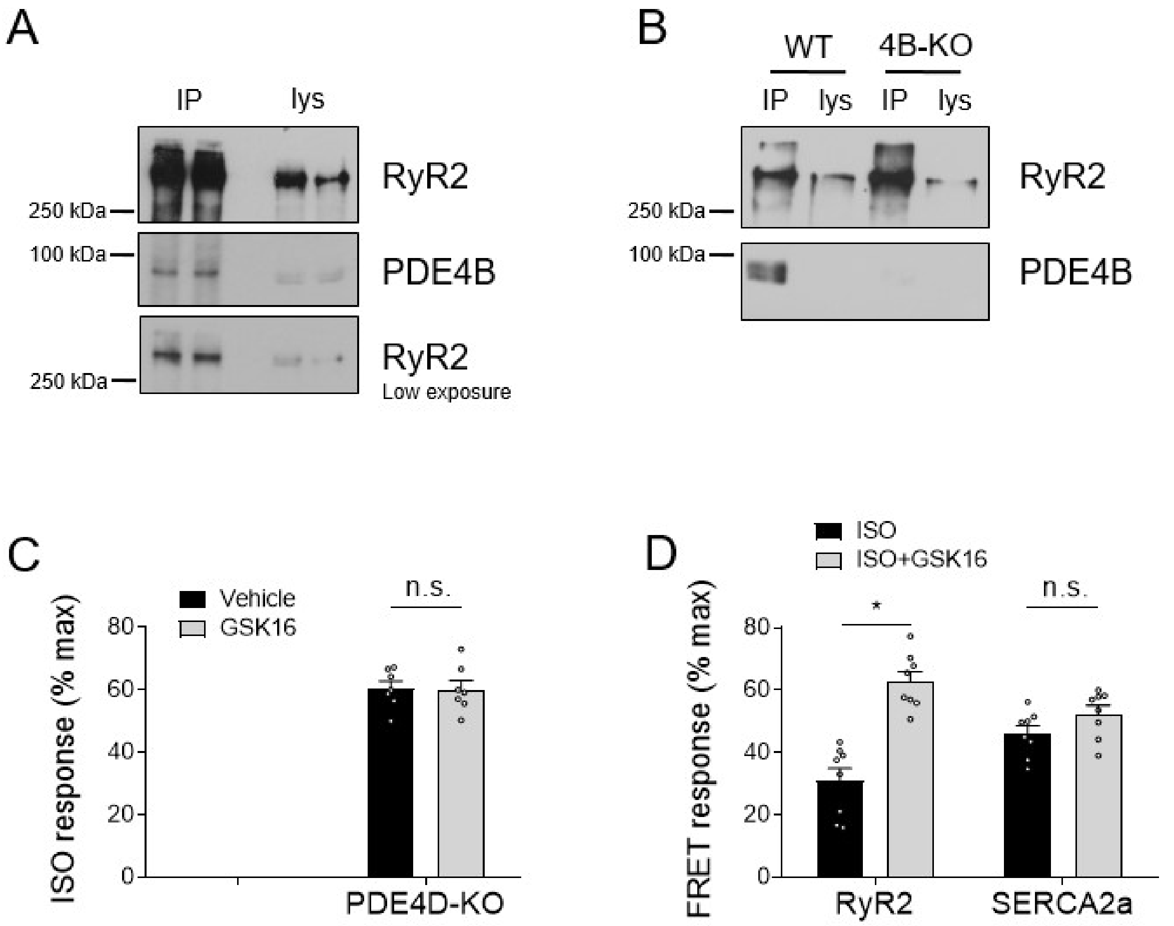

Supplementary Figure S2. PDE4B co-immunoprecipitates with endogenous cardiac RyR2. Wildtype heart lysates were immunoprecipitated using total RyR2 antibody, and IP fractions were probed for PDE4B. (**B**). Control experiment performed using PDE4B-KO hearts. Representative immunoblots for n=4 hearts are shown. The results of the same experiment performed under identical conditions for PDE4D have been previously reported, please see Ref. 27. (**C**). Analysis of ISO response amplitude from FRET measurements performed in PDE4D-KO CMs as described in Figures 2A,B pretreated for 5 min either with vehicle of with 100 nmol/L of the selective PDE4B inhibitor GSK16. n=7 cells each from two independent isolations, n.s. – not significant by mixed ANOVA followed by Wald x^2 test. (**D**) Analysis of ISO and ISO+GSK16 response amplitudes from FRET measurements shown in Figure 4E. * $p<0.05$, n.s. – not significant by mixed ANOVA followed by Wald x^2 test.

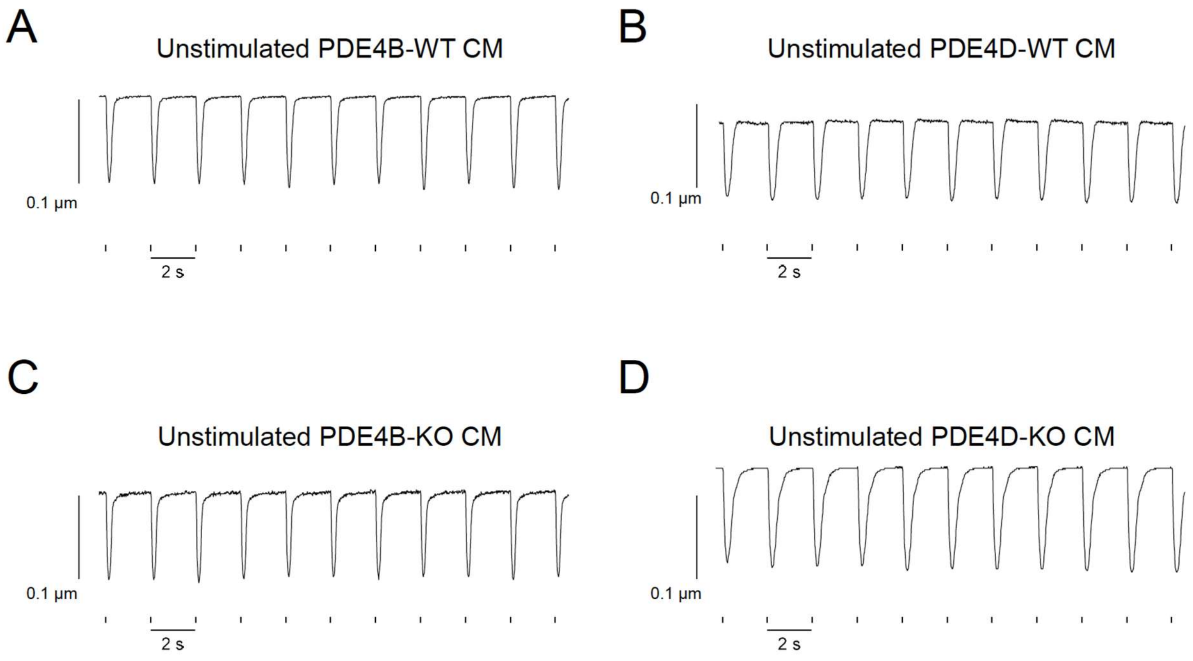

Supplementary Figure S3. Measurements of arrhythmia susceptibility in isolated myocytes. (A-D) Representative traces for untreated CMs paced for 4 min at 0.5 s^{-1} and 15.0 V. Quantification of extra beats and n numbers are presented in Figure 5E.

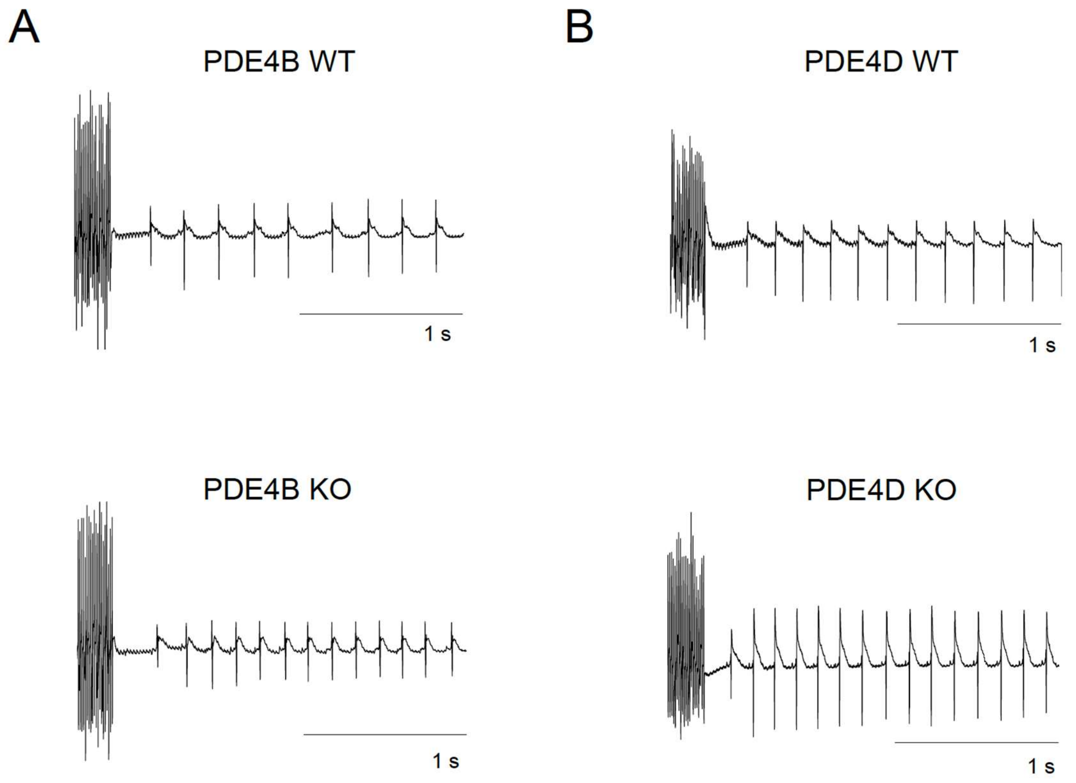

Supplementary Figure S4. Arrhythmia occurence in Langendorff perfused hearts. Hearts were equilibrated for 15 min and measured as described in Figure 6 and in Methods without ISO stimulation. Shown are representative right ventricular electrograms from PDE4B WT vs. PDE4B KO (**A**) and PDE4D WT vs. PDE4D KO hearts (**B**). Quantification and n numbers are presented in Figure 6B/D.

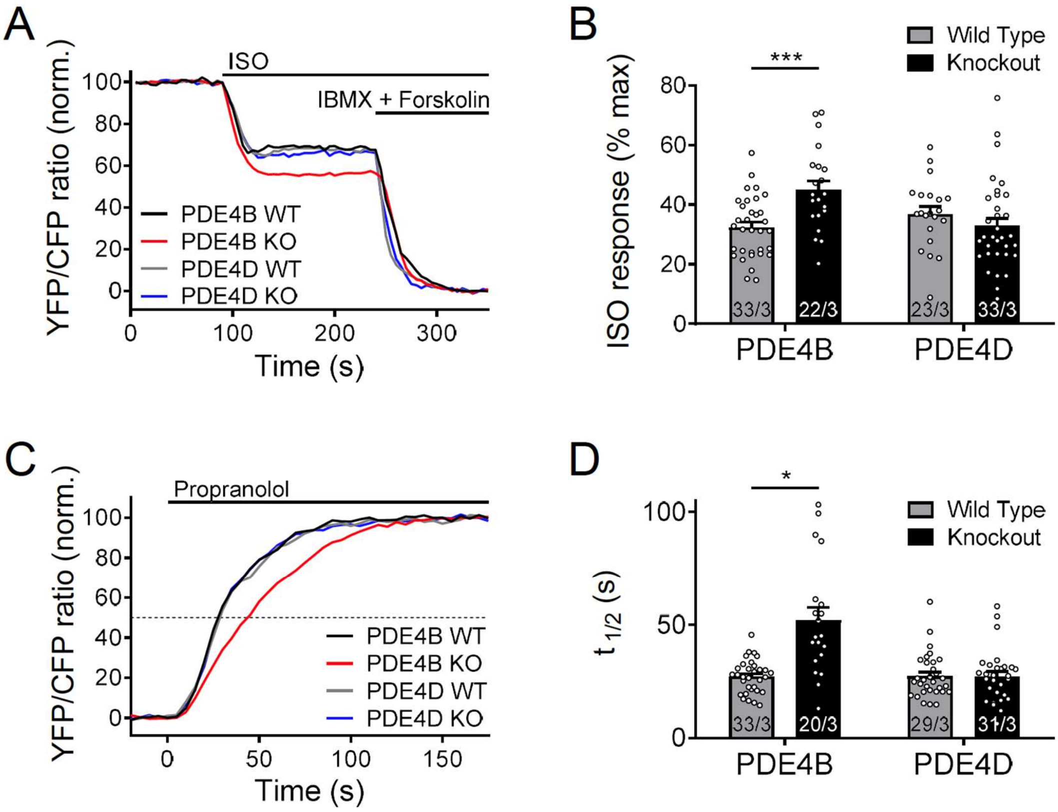

Supplementary Figure S5. PDE4B regulates cAMP signaling events in the RyR2 microdomain of chemically detubulated cardiomyocytes (treatment with 1.5 mol/L formamide for 15 min). FRET experiments with adult mouse cardiomyocytes (CMs), freshly isolated from PDE4B-WT, PDE4B-KO, PDE4D-WT and PDE4D-KO mice harboring the RyR2 microdomain specific FRET biosensor Epac1-JNC. **(A)** Averaged FRET traces from n cells/N hearts (33/3 for PDE4B WT, 22/3 for PDE4B KO, 23/3 for PDE4D WT and 33/3 for PDE4D) from formamide detubulated CMs stimulated with 100 nmol/L Isoprenaline (ISO) followed by 100 µmol/L 3-isobutyl-1-methylxanthine (IBMX) and 10 µmol/L forskolin. **(B)** Quantification of ISO response compared to maximal cAMP response in detubulated CMs. **(C)** Averaged FRET traces (33/3 for PDE4B WT, 20/3 for PDE4B KO, 29/3 for PDE4D WT and 31/3 for PDE4D KO) from 100 nmol/L ISO prestimulated formamide detubulated CMs treated with 100 µM propranolol. **(D)** Quantification of $\tau_{1/2}$ for propranolol responses in detubulated CMs. Data are from n cells / N mice are presented as means ± SEM. *, *** - significant differences at $p<0.05$, $p<0.001$, mixed ANOVA followed by Wald x^2 test.

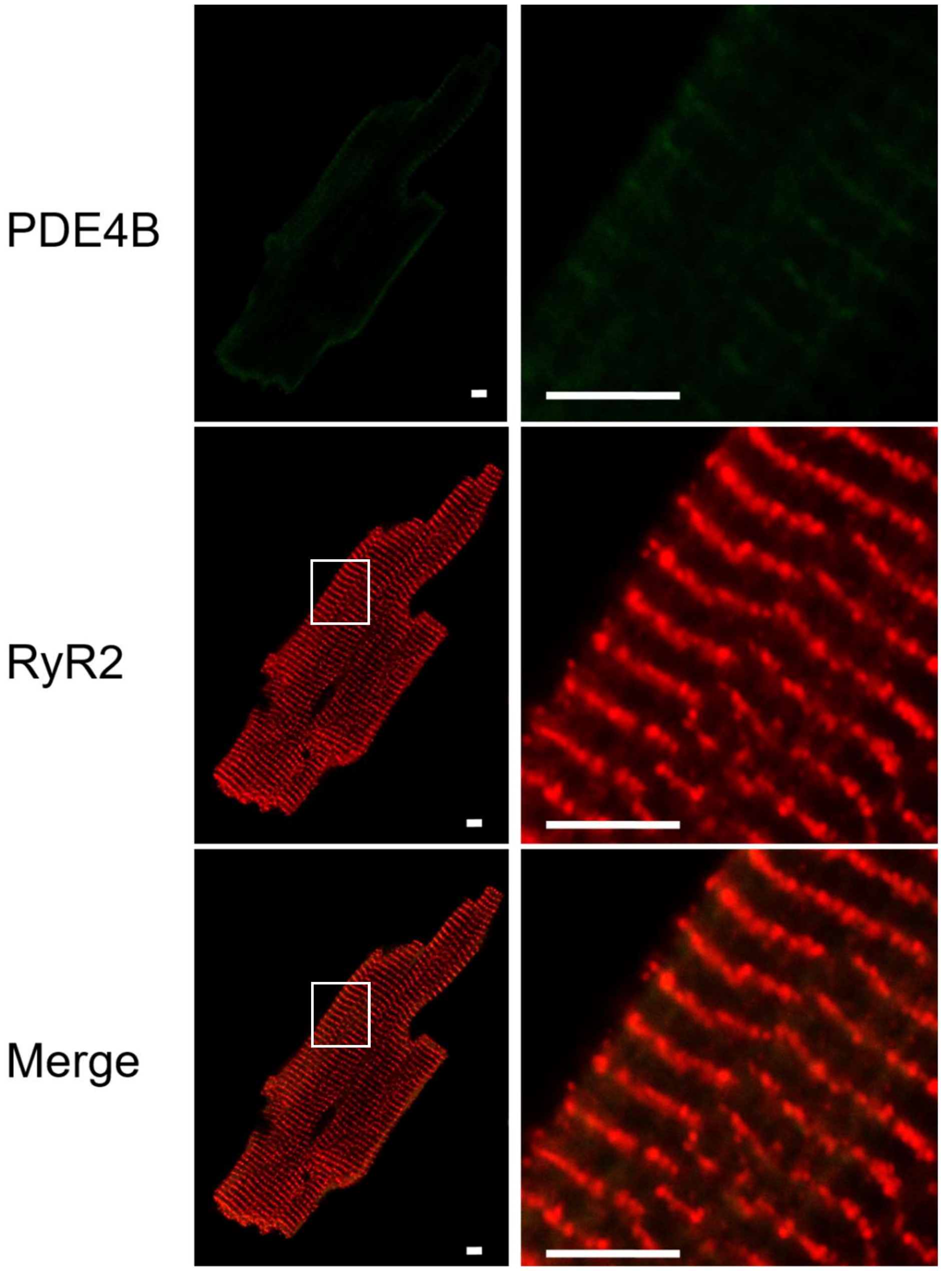

Supplementary Figure S6. Negative control of PDE4B immunostaining for the experiment shown in Figure 7. Staining and confocal images were performed using PDE4B-KO CMs and exactly the same settings as described in Figure 7 and in Materials and Methods. Representative images (n=10), scale bars, 5 µm.
